# Pushing Structural Information into the Yeast Interactome by High-Throughput Protein Docking Experiments

**DOI:** 10.1371/journal.pcbi.1000490

**Published:** 2009-08-28

**Authors:** Roberto Mosca, Carles Pons, Juan Fernández-Recio, Patrick Aloy

**Affiliations:** 1 Institute for Research in Biomedicine, Barcelona, Spain; 2 Barcelona Supercomputing Center, Barcelona, Spain; 3 National Institute of Bioinformatics (INB), Barcelona, Spain; 4 Institució Catalana de Recerca i Estudis Avançats (ICREA), Barcelona, Spain; Stanford University, United States of America

## Abstract

The last several years have seen the consolidation of high-throughput proteomics initiatives to identify and characterize protein interactions and macromolecular complexes in model organisms. In particular, more that 10,000 high-confidence protein-protein interactions have been described between the roughly 6,000 proteins encoded in the budding yeast genome (*Saccharomyces cerevisiae*). However, unfortunately, high-resolution three-dimensional structures are only available for less than one hundred of these interacting pairs. Here, we expand this structural information on yeast protein interactions by running the first-ever high-throughput docking experiment with some of the best state-of-the-art methodologies, according to our benchmarks. To increase the coverage of the interaction space, we also explore the possibility of using homology models of varying quality in the docking experiments, instead of experimental structures, and assess how it would affect the global performance of the methods. In total, we have applied the docking procedure to 217 experimental structures and 1,023 homology models, providing putative structural models for over 3,000 protein-protein interactions in the yeast interactome. Finally, we analyze in detail the structural models obtained for the interaction between SAM1-anthranilate synthase complex and the MET30-RNA polymerase III to illustrate how our predictions can be straightforwardly used by the scientific community. The results of our experiment will be integrated into the general 3D-Repertoire pipeline, a European initiative to solve the structures of as many as possible protein complexes in yeast at the best possible resolution. All docking results are available at http://gatealoy.pcb.ub.es/HT_docking/.

## Author Summary

Proteins are the main perpetrators of most biological processes. However, they seldom act alone, and most cellular functions are, in fact, carried out by large macromolecular complexes and regulated through intricate protein-protein interaction networks. Consequently, large efforts have been devoted to unveil protein interrelationships in a high-throughput manner, and the last several years have seen the consecution of the first interactome drafts for several model organisms. Unfortunately, these studies only reveal whether two proteins interact, but not the molecular bases of these interactions. A full comprehension of how proteins bind and form complexes can only come from high-resolution, three-dimensional (3D) structures, since they provide the key quasi-atomic details necessary to understand how the individual components in a complex or pathway are assembled and coordinated to function as a molecular unit. Here, we use protein docking experiments, in a high-throughput manner, to predict the 3D structure of over 3,000 interactions in yeast, which will be used to complement the complex structures obtained within the 3D-Repertoire pan-European initiative (http://www.3drepertoire.org).

## Introduction

In the last decade, many genome-sequencing projects started delivering nearly complete lists of the macromolecules present in several model organisms. However, taken individually, knowing the components reveal relatively little about how complex systems, such as a eukaryotic cell, assemble and coordinate the many discrete functions needed for its correct functioning. Most cellular processes are carried out by large macromolecular complexes and regulated through a complex network of transient protein-protein interactions, defining the *Interactome* of a given organism. Accordingly, the last years have seen the emergence of many high-throughput proteomics initiatives devoted to the identification of new protein interactions and macromolecular complexes in model organisms [1]–[6], including human [7],[8]. These efforts have developed mostly around two different techniques: the yeast two-hybrid system, more suitable for identifying binary interactions, and affinity purifications coupled to mass spectrometry analyses, for discovering multi-protein assemblies. Taken together, they have unveiled thousands of new unsuspected interactions, which are now properly stored and classified in public databases [9], and have changed the way biologists approach complex cellular functions, setting the ground for systems biology [5].

However, these techniques can only identify whether two proteins interact or the composition of molecular complexes and, in the best cases, which are the individual domains mediating the interaction. A full comprehension of how proteins bind and form complexes can only come from high-resolution three-dimensional (3D) structures, since they provide the atomic details necessary to understand how the interactions occur and the high degree of specificity observed can be achieved [10].

Unfortunately, despite the efforts of ongoing structural genomics (SG) projects to extend the structural coverage of the sequence space for the proteome of several organisms, it seems that structural biology is somehow lagging behind the new trends in high-throughput biology. In fact, since the first genome-wide interaction discovery experiments were published, there has been an increasing gap between the number of identified interactions and those for which their 3D structure is known [11]. It is thus crucial to come up with effective strategies to incorporate structural information into interactome networks. Indeed, we belong to a pan-European venture, the 3D-Repertoire project, which aims at solving the structures of all amenable protein complexes in yeast at the best possible resolution (http://www.3drepertoire.org). The 3D-Repertoire consortium will attempt to experimentally solve the structure of some 100 yeast complexes by means of X-ray crystallography, nuclear magnetic resonance (NMR), electron microscopy (EM) or a combination of these techniques. However, the vast majority of complexes and interactions will be tackled with computational methods in combination with low resolution structural data (e.g. low-resolution EM or small-angle x-ray scattering (SAXS)). The first step in the structural bioinformatics pipeline that we have established within the consortium is to model by homology as many yeast interactions as possible, in the same way that we can model individual proteins. This is certainly possible, since it has been shown that most interologues (i.e. homologous interacting pairs) do indeed interact in the same way [12]. These models will then be complemented with low-resolution structural information, whenever it is available, to build the most complete possible models [13]. However, unfortunately, interaction templates are only available for a very limited number of interactions and thus, to get a more complete picture of the yeast interactome, it is necessary to apply methodologies that are template-independent.

Computational docking aims to predict the structure of a complex formed by two interacting proteins starting from the structures of the individual components. Many different docking methods have been reported, with increasing success rates (see [14]–[16] for a review). However, given the number and variety of available docking methods, the community found it desirable to validate and compare them in a blind contest. The recent CAPRI experiments (http://www.ebi.ac.uk/msd-srv/capri/) provide an objective assessment of current docking methods and their successes and limitations [17]–[19]. The majority of the most popular docking methods are based on a rigid-body approach (i.e. they do not allow backbone flexibility), and can be roughly classified in two types: i) those that focus on exhaustive sampling in search for geometric surface correlation (mainly through FFT -Fast Fourier Transform-, or geometric hashing algorithms), and ii) those that place more emphasis on energy-based sampling (usually by minimization, molecular dynamics or Monte-Carlo) and/or scoring. Two representative FFT-based methods of the first type are FTDock [20] and ZDOCK [21],[22], in its several versions of increasing complexity and prediction accuracy [23]. Other successful geometric-based docking methods are Hex [24] or MolFit [25]. On the other hand, energy-based sampling and scoring schemes have also been evaluated in the CAPRI experiment. For instance, methods like ICM-DISCO [26], which used a Monte Carlo rigid-body search on grid-based potentials with an essential evaluation step based on electrostatics and desolvation [27],[28] were very successful in the first two CAPRI editions. This evaluation scheme was recently implemented in pyDock [29] to permit the rescoring of docking sets generated by other independent methods, which yielded top results as scorer tool in the most recent CAPRI meeting [30]. Other methods do also successfully apply energy evaluation during or after the docking generation phase, like Haddock [31], ClusPro/SmoothDock [32],[33], RosettaDock [34], or ATTRACT [35].

However, despite the improvement in docking methods, it is still difficult to know in advance whether the predicted binding modes will be close to the real interaction topology or not. The CAPRI initiative has identified the large conformational changes upon association as the best measure for assessing the difficulty of docking experiments [36],[37], but these changes cannot be foreseen before the experimental structure of the complex is available and thus have very limited predictive value.

In this work, we test the performance of two of the best docking programs in the market (FTDock and ZDOCK), together with one of the most successful docking scoring schemes (pyDock), against the most recent and comprehensive benchmark set available [38]. We then use the results of the benchmark to explore the possibility of setting a general confidence threshold for docking scores to increase the reliability of the predictions. In addition, we also assess how the use of homology models of varying quality in docking experiments, instead of experimental structures, would affect the global performance of the methods. Finally, we apply all the gained knowledge to run the first ever high-throughput docking experiment, which provides putative models for over 3,000 protein-protein interactions in the yeast interactome.

## Methods

### Docking methods and parameter selection

We generated a collection of docking solutions based only on geometry complementarity by running FTDock [20] under the standard conditions recently reported [29] (i.e. no electrostatics, 1.2 Å grid size, 12° angle resolution). In addition, we also tested two different versions of ZDOCK that include additional functions in the FFT-based correlation, with expectedly better success. ZDOCK 2.3 [21] combines pairwise shape complementarity [39] with desolvation calculations based on atomic contact energies [40] and Coulombic electrostatics [22]. ZDOCK 3.0 [23] is a novel and improved version that replaces the simplified averaged atomic contact energies with atomic pairwise statistical potentials using an optimized atom type alphabet [41]. We used default parameters on all the versions of ZDOCK tested. Additionally, we applied pyDock [29] to re-score the sets of rigid-body solutions provides by each docking program. The pyDock scoring function is composed of Coulombic electrostatics with distance-dependent dielectric constant, ASA-based desolvation with atomic solvation parameters previously optimized for rigid-body docking, and van der Waals energy (with 0.1 weighing factor, and truncated to +1.0 kcal/mol to allow certain overlap of the structures). This scoring function was shown to be the best for several targets of the CAPRI experiment [30]. In this work, we tested the use of pyDock with and without the van der Waals energy term. Before applying the different docking procedures, the coordinates of each x-ray structure were automatically checked with the pyDock module “setup”, re-building incomplete sidechains with SCWRL 3.0 [42] and removing missing backbone atoms (usually incomplete N-terminal or C-terminal residues). We also excluded cofactors, ions and other heteroatoms from docking and scoring calculations.

### Benchmark set

To assess the accuracy of the docking methods used in the study, we used the most recent, and well-accepted, benchmark set of protein-protein interactions [38] (Benchmark3.0). We also used the same benchmark set to identify a confidence threshold on the score assigned by the docking programs. This set consists of 124 docking non-redundant cases, for which high-resolution crystal structures are available for both the bound complex and for the single unbound components. Docking experiments are run on the unbound structures and the results evaluated by comparing them to the solved structure of the bound complex. The dataset is non redundant in the sense that it does not contain interactions that share the same family-family class in Pre-SCOP (http://www.mrc-lmb.cam.ac.uk/agm/pre-scop/). Test cases presenting more than two missing residues in the interface or presenting different cofactors at the binding site between the bound and unbound structures are excluded from the dataset. The Benchmark3.0 also classifies the 124 cases based on their level of docking complexity into Rigid body (88), Medium (19) and Difficult (17). The three levels span a large variety of interaction types including enzyme-inhibitor, antigen-antibody and other types of transient interactions.

### Evaluation of the docking poses

We assessed the quality of the solutions provided by the different docking programs using the same criteria that are used in the CAPRI experiment [30]. Of the two docked structures one is conventionally called the *receptor* (usually the biggest) and the other is called the *ligand* (usually the smallest). Docking solutions are then classified as Incorrect, Acceptable, Medium and High based on the RMSD between the bound and unbound ligands after superposition of the receptor, the RMSD of the interface and the number of conserved/non-conserved native interactions. Details on the method used to calculate the classification can be found in Méndez et al. [18]. In this case, we did not apply the CAPRI filter to remove solutions presenting an excessive number of clashes. It is worth noting that, in contrast to the ligand RMSD evaluation strategy used in the CAPRI experiment, we did not apply any filter to remove from the calculation those parts of the structures that do not move as rigid bodies (turns and small loops).

### Selection of candidates for the high-throughput docking experiment from the yeast interactome

First of all, we would like to stress that our goal is not to predict interactions between yeast proteins, but to provide putative models of those interactions that have already been experimentally determined. Thus, the first step towards predicting the structure of yeast complexes was to identify and compile all the available structures for the individual protein components. We started by downloading all the sequences for the systematically named ORFs in the Saccaromyces Genome Database (SGD, ftp://ftp.yeastgenome.org/yeast/, [43]) as of September 2008. We excluded dubious ORFs and pseudogenes and eliminated duplicated ORFs from the dataset. We then used the yeast ORF sequences to search the space of known high-resolution three-dimensional (3D) structures in the Protein Data Bank [44] (PDB, www.pdb.org) using BLAST [45]. To infer the 3D structure of a given ORF we required a BLAST E-value ≤1e-4, a sequence identity ≥98% and a coverage ≥90%. NMR structures and PDB files including multiple models of the same structure were discarded. For all the sequences for which it was not possible to find a complete structure, we searched ModBase [46] (http://salilab.org/modbase) for homology models. We retained all the models with more than 30% sequence identity, spanning more than 90% of the ORF length and having a score higher than 0.7. For every ORF with multiple models, we selected the one with the highest sequence identity as a representative. For all the sequences without experimental structures and complete homology models, we retained partial models (having less than 90% coverage), provided that they were spanning at least 90% of one PFAM [47] domain as identified on the sequence of the original ORF. For the domains, we always kept the longest model spanning that domain (i.e. the one with the best coverage). For some of the ORF we collected multiple partial models. Table 1 summarizes the results of the collection of yeast protein structures.

**Table 1:** Summary of the high-throughput docking experiment.

**Number of initial ORF sequences**	**5821**
**Number of sequences with a corresponding structure or model (in interactome)**	**1892 (1240)**
Number of sequences with an experimental structure (in interactome)	336 (217)
Number of sequences with a complete model (in interactome)	441 (249)
Number of sequences with a partial model (in interactome)	1115 (774)
**Total number of high confidence interactions**	**13614 ORF pairs**
**Number of interactions with structural data for the interacting proteins**	**3091 ORF pairs (3711 interactions)**
Number of interactions with experimental structure	91 ORF pairs
Number of interactions modeled by homology	135 ORF pairs
**Number of successful docking experiments…**	**3401**
…between experimental structures	325
…involving complete models or experimental structures	348
…involving partial models	2728

Once identified all those yeast proteins for which we know the 3D structure, or can model it, of at least one domain, we need to compile all those protein-protein interactions and complexes that have been experimentally identified in yeast (Table 2). We took directly inferred binary interactions for those coming from two hybrid experiments, and used a SPOKE expansion (i.e. the bait against every prey) whenever the interacting partners were discovered through affinity purification techniques. We used a MATRIX expansion (i.e. all against all) for protein complexes. We also merged interactions from Intact [48] and MINT [49] and selected only those ones that were confirmed by either more than one source, more than one method or by x-ray crystallography. For pairs of interacting ORFs having multiple partial models we run docking experiments on all the possible pairs. We also identified all those interactions that either had a known 3D structure already deposited in the PDB (termed *experimental structures* of the interacting protein pair) or that could be modelled by homology (see Text S1 the Supplementary materials for details on how interactions between chains were identified). To find the structural templates for homology modelling we searched the PDB and looked for protein chains homologous to the yeast ORFs involved in our interaction set, excluding those for which an experimental structure is available. We considered only those with a BLAST E-value ≤1e-4, a coverage ≥90% and a sequence identity ≥30%. We then matched the hits found with our set of interacting pairs. The interactions were modeled by superposing the structure or model of the interacting partners to the corresponding structure of the homologous protein in the template. Alignments and superpositions were performed using RAPIDO [50] with default parameters and selecting the rigid superposition. We also applied an additional filter to exclude models of poor quality (presenting strong incompatibilities, like large clashing areas or poor structural alignments between the original structures and the template). See Text S1 in the Supplementary materials for details on the filtering procedure.

**Table 2:** Sources of experimentally identified protein-protein interactions in yeast.

Description	Reference	URL	Interactions
**High-Quality Binary Protein Interaction Map of the Yeast Interactome Network**	Yu et al. 2008 [62]	http://interactome.dfci.harvard.edu/S_cerevisiae/	1809
**WI-PHI** (High confidence interactions having a score greater than 21, corresponding to the “WI-PHI core” dataset)	Kiemer et al. 2007 [63]	ftp://mint.bio.uniroma2.it/pub/wifi/	5299
**Proteome survey reveals modularity of the yeast cell machinery** (All binary interactions with a socio-affinity score greater than 10 were included)	Gavin, Aloy et al. 2006 [5]	http://yeast-complexes.embl.de/	1645
**MINT +**	Chatraryamontri et al. 2006 [49]	http://mint.bio.uniroma2.it/mint	10098
**Intact**	Hermjakob et al. 2004 [48]	http://www.ebi.ac.uk/intact	
**Total**			**13614**

### Collection of models for the Benchmark 3.0

To assess the validity of running docking methods on homology models, rather than on experimentally determined structures, we collected models for each protein in Benchmark 3.0 from ModBase [46] (http://salilab.org/modbase). We selected the models based on two criteria: the template used had more than 30% and less than 98% sequence identity to the target, and the score of the model was higher than 0.7. Using these criteria, we finally picked 283 models for 75 of the 248 single proteins in Benchmark 3.0 (receptor and ligand for all the 124 cases). For many of the proteins several models were available based on different templates. It is known that the structural similarity of a model to the real target is affected by the sequence identity of the template to the target protein [51]. For this reason we randomly generated different sets of models (in every set one model was selected for each protein) in such a way that the distribution of the sequence identity of the templates in every one of the sets corresponded to the distribution of the sequence identity observed in the set of models selected for the large scale docking experiment (Figure S1 in the Supplementary materials). For it to be possible every set had to be composed by no more than 56 models. For every one of those subsets we calculated the average RMSD between the models, the bound and the unbound structures. A plot of the distribution of the three average RMSDs (model/bound, model/unbound, bound/unbound) is shown in Figure S2, in the Supplementary materials. Finally we selected a set of complete cases (for which there were models both for the receptor and the ligand) with a distribution of the sequence identity corresponding to the one observed in the models from the large scale experiment. This was possible for 13 out of 124 cases. For those cases we ran ZDOCK 3.0 on the model to predict the structure of the binary complex and we evaluated the resulting predictions by comparing them with the crystallographic structure of the complex.

### Classification of interactions into binary and multi-component

We considered as *binary* interactions those that involve only two proteins and have been identified by one of the following techniques: array technology, cross-linking study, cytoplasmic complementation assay, nuclear magnetic resonance, two hybrid or x-ray crystallography.

Alternatively, we considered an interaction as *multi-component* if both the interacting proteins belong to the same aggregate in a list of multi-component aggregates generated by merging data extracted from MPACT [52], MINT [49] and Intact [48]. We collected all the known complexes in yeast from MPACT and added them to the list together with all the interactions involving more than two proteins and reported in one of the following publications about large scale experiments using tandem affinity purification techniques: Gavin et al. 2002 [53], Ho et al. 2002 [54], Krogan et al. 2004 [55], Gavin et al. 2006 [5], Krogan et al. 2006 [6].

## Results/Discussion

### Selection of the most appropriate docking strategy for the high-throughput experiment

The first step in this study is to thoroughly benchmark some state-of-the-art docking strategies and decide which one is the optimal to approach our high-throughput docking experiment in yeast. To carry out this first task, we selected the most recent and well-accepted benchmark set for protein docking developed in the Zlab laboratory [38] (http://www.zlab.bu.edu). As explained in the *Methods* section, this dataset consists of 124 interacting pairs for which a high-resolution structure of the complex and the individual components exist. We generated ranked docking poses for the 124 interactions in the benchmark set with FTDock [20], ZDOCK 2.3 [21] and ZDOCK 3.0 [23]. We then rescored the docking solutions generated by these three programs with pyDock [29]. We selected these docking programs because they are among the ones having the best performance in the last rounds of the CAPRI experiment [17] and also for their availability as standalone programs, which makes them suitable for a large scale docking experiment. It is important to note that the programs used in the test only produce rigid body solutions, meaning that no conformational change is introduced in the interacting molecules. In more standard applications of docking programs to individual cases, there is usually the possibility of integrating biological knowledge (i.e. site directed mutagenesis studies) on the interacting interface, model conformational changes and flexibility and to perform several iterations of refinement to remove impossible solutions and improve the quality of the remaining. However, unfortunately, this is not feasible in our study due to the large number of docking experiments and the computational cost of the refinement step, which forces us to assess the accuracy of the docking solutions as they come out of the programs, without applying any further biological filtering.

**Figure 1:**
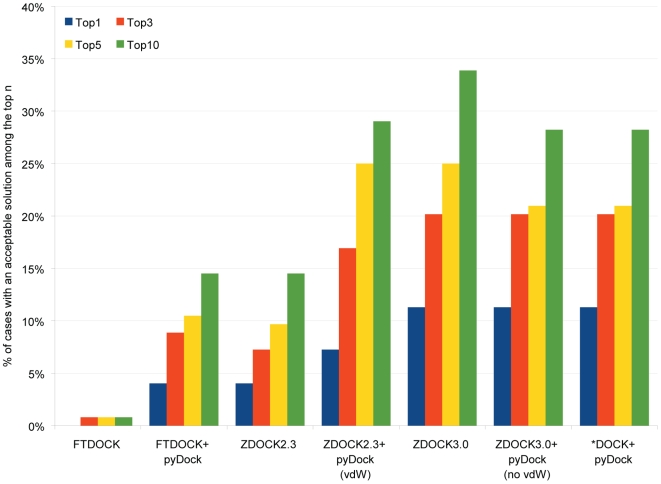
Percentage of cases with an acceptable solution ranked in the top positions. Percentage of cases for which an “at least acceptable” solution is found in the top n (n = 1,3,5,10) for the different docking tools that have been tested.

Figure 1 shows the results of the benchmark. ZDOCK 3.0 and ZDOCK 3.0+pyDock are the two methods having the best performance. By using one of them it is possible to obtain an acceptable solution among the top 3 for roughly 20% of the cases (see also Table S1 in the Supplementary materials). If we consider only the top solution for each interacting pair, we find an acceptable solution for 14 out of the 124 cases tested. This figure goes up to 25 if we contemplate an acceptable solution in the top 3 and to 42 when considering the top 10 solutions generated by ZDOCK 3.0. The results of FTDock and ZDOCK 2.3 both, individually and with the rescoring provided by pyDock, are clearly outperformed by ZDOCK 3.0 and ZDOCK 3.0+pyDock, which have similar success rates on the top 3 solutions (even if in different cases), with ZDOCK 3.0 showing a few more successful cases in the top 5 and 10 solutions. We also explored the possibility of merging and re-scoring the results provided by the different programs which, unfortunately, did not improve the results (Figure 1). Thus, in light of the obtained results, we proceeded to the next steps using only ZDOCK 3.0 and pyDock, excluding FTDock and ZDOCK 2.3.

It is important to highlight that many of the correct predictions are classified as “rigid body” docking cases in the Benchmark 3.0 dataset. These are the interaction pairs that do not undergo important conformational changes upon association and thus it is easier to find a good docking solution starting from the unbound states. Table S5 (in the Supplementary Materials) shows the distribution of good cases (i.e. cases having at least one acceptable solution among the top *n*) between the different categories of difficulty as reported in Benchmark 3.0.

### Improving the accuracy by setting a score threshold

There is evidence that the raw scores provided by docking methods often show a poor correlation with the probabilities of a given solution to be correct and, perhaps more importantly, these scores are not comparable between experiments involving different molecules, since they are very much dependent on the size and shape of the molecules tested [56]. However, given that we are benchmarking state-of-the-art methods on a large set of protein interactions, and that we need to drastically reduce the number of solutions to be included in the 3D-Repertoire modelling pipeline, we decided to explore the possibility of increasing the accuracy of the results by identifying a general score threshold, at expenses of reducing the coverage. This is to reject those results that are more likely to be incorrect and to keep the ones that have a higher probability of being acceptable predictions of the real interaction. Our aim, in fact, is to select a small subset of cases on which we can have higher confidence about the correctness of the generated predictions.

After several trials, we found that by imposing a threshold on the average score of the top 3 solutions we could improve the success rate of a 10%, going from 20% to almost 30% of successful cases.

**Figure 2:**
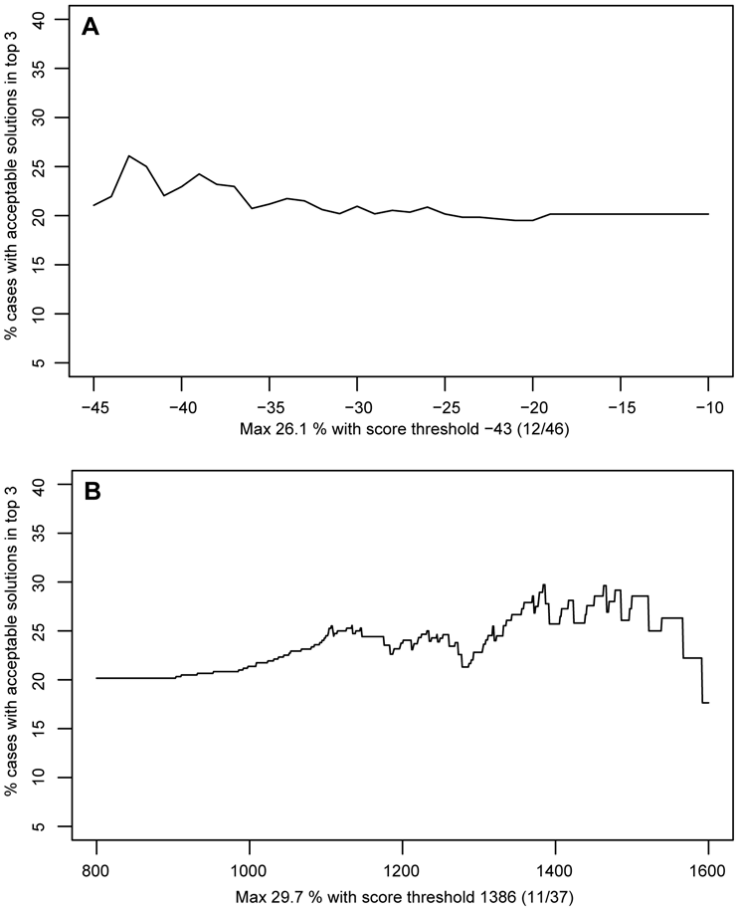
Success rate by using a threshold on the score. Ratio between the number of “good” cases (cases having at least one acceptable solution in the top 3) and the total number of cases satisfying the threshold for increasing values of the threshold. (A) refers to ZDOCK 3.0+pyDock (without Van der Waals contribution) while (B) refers to ZDOCK 3.0 alone.

In particular, we analysed the ratio between the number of good cases and the number of total cases satisfying the threshold. It is important to note that while the score produced by pyDock is minimized the one produced by ZDOCK is maximized. Thus in the case of pyDock a case is selected if the average score of the top n solutions is lower than the threshold while for ZDOCK the average score must be higher then the threshold. The analysis was repeated for the top 1, 3, 5 and 10 solutions for both the programs and for different values of the threshold (see Figure S2 in the Supplementary materials). The analysis shows that there is no defined trend in the success rate for the selected cases. The plots are not strictly monotonic but they show a moderate degree of variation for increasing values of the threshold. Nevertheless in all the cases the threshold shows a certain degree of success in filtering out cases showing no acceptable solution, increasing in this way the accuracy for the selected ones. We picked as the best result the one produced by ZDOCK 3.0, without the rescoring provided by pyDock, on the top 3 solutions (Figure 2), corresponding to a score threshold of 1386, which results in an increase of the accuracy from 20% (25 good cases on 124 without the threshold) to 29.7% (11 good cases over the 37 selected).

We would like to stress that the success rates obtained in our high-throughput strategy are in good agreement with the average success rate of the best predictors in the CAPRI experiment (see Text S1, Figure S4 and Table S6 in the Supplementary materials), confirming that our fully automated results do indeed match the state-of-the-art of the docking field. We have also explored the added value of expert manual intervention in specific docking predictions, and discovered that it represents an improvement in the capacity of docking to produce accurate models of about 8% on average.

The choice of the top 3 cases was taken as a compromise between the increased accuracy and the number of predictions to be analysed for every case. In fact, even if it would be desirable to have just one correct prediction for every case, taking only the top first solution yields to a probability of success significantly lower (18% in the best case). On the other hand, considering the top 10 solutions would raise the accuracy to the highest success ratio (38.5%) but it would lead to an explosion in combinatorial complexity of the model building procedure within the 3D-Repertoire pipeline. In the attempt to build higher order structures (i.e. larger subcomplexes) from binary interactions, we assemble all possible combinations of binary structures and assess their fit by, for instance, computing the number of clashes and the binding energies by means of empirical force fields and testing the fit of available experimental information. This is a very time consuming process that grows exponentially with the number of complex components (14 on average for our set of complexes). It is thus unfeasible to test many possibilities for each binary interaction, and that is why we have reduced the number of docking solutions kept for further exploration to three which, we think, it is a good accuracy/coverage compromise.

Although the overall results, in terms of accuracy, are very similar between ZDOCK 3.0 and ZDOCK 3.0+pyDock, the interaction pairs that each method correctly identifies are not the same (see Table S2 in the Supplementary materials). A deeper investigation of the differences between the successful cases of the two programs could help the further development of the docking methods themselves. As there is no general agreement between the two on the good cases, we could not use this information to improve the accuracy in the selection of successful docking cases.

Unfortunately, and despite the success achieved by the many ongoing structural genomics efforts, the availability of high-resolution structures for most proteins is still very limited. Thus, as for individual structures, a good strategy to increase the coverage of the structural space is to build models by homology [57] and, as described in the methods section, many of the individual structures that we used in the high-throughput docking experiment in yeast are, in fact, homology models. Consequently, on the one hand we need to test whether the level of accuracy in docking experiments is similar to the one achieved when experimental structures are used and, on the other, if the score thresholds derived from experimental structures are still valid for docking homology models. The ideal test would be to generate homology models for the 124 protein interacting pairs in the benchmark set using structural templates in the range of sequence identities similar to those used for modelling the yeast proteins and, obviously, discarding the real structures. Unfortunately, we could build homology models, in the same fashion as that used in the high-throughput experiment, for both individual structures of only 13 of the interacting pairs. Of these, 5 passed the score threshold and in only one case we found an acceptable solution among the top 3, which would represent an accuracy of 20%, somewhat below the almost 30% achieved when using experimental structures. However, it is clear that we have too few cases to extract any relevant conclusion, which prompted us to look for alternative ways of assessing the use of homology models and score threshold in docking experiments.

The success of docking experiments largely depends on the structural conformational changes that the two protein components suffer upon association. In other words, when the unbound and bound forms of the interacting proteins are similar, it is very likely to obtain docking solutions of high-quality and, when the two proteins undergo severe conformational changes, it is almost impossible to get any acceptable solution. Consequently, we could compare the structural differences between the unbound/bound protein forms and models/bound forms in the benchmark set to estimate the validity of using homology models in docking experiments (see the *Methods* section for details as to how we selected the models).

For the interacting protein pairs in the benchmark set, we observed that the difference between the models and the bound structures is, in general, a bit higher than the difference between the unbound and bound structures (Figure S3 in the Supplementary materials) suggesting that the docking procedure would yield a lower success rate when using models instead of the experimental structures. Nevertheless for more than 50% of the cases the difference between the RMSDs of models/bound and unbound/bound is very little (less than 1 Å). For 101 cases the conformational changes are more pronounced between the models and the bound forms than for the unbound/bound pairs. However, interestingly, there are 23 models that are more similar to the bound structures than the corresponding unbound experimental forms, suggesting that for those cases the docking experiment might have higher probability of success by using the models. Overall, our analyses show that it is indeed reasonable to run docking experiments using homology models of the individual proteins, but it is likely that it can decrease the success rate of the experiments although it is difficult to quantify its real impact.

### High-throughput docking experiment

The starting point of our experiment was a high-confidence set of 13614 protein-protein interactions in yeast obtained by merging the biological data contained in the different available databases (Table 1). We then collected structural data, in the form of experimental structures or homology models, for the proteins involved in the interactions (see *Methods*). We found experimental structures for 217 of the proteins in the high-confidence interactome, while for another 249 proteins we could collect a complete model built by homology. For the remaining 774 proteins we only obtained partial models, corresponding mainly to individual domains. This is not a problem, since it has been shown that in the vast majority of the interactions between multi-domain proteins only one domain in each protein is directly involved in the interaction [58]. For some of the proteins we found more than one partial model, representing different domains in the polypeptidic chain. It is interesting to note that 10 of the proteins for which we have a complete experimental structure and the structural templates used to build 173 of the homology models were solved within SG initiatives. While this illustrates the impact of SG projects, which have substantially improved (around 20%) the coverage of the yeast sequence space in about 20%, providing a template for a significant number of proteins, the number of sequences for which we have neither a structure nor an available template for modeling is still high.

**Figure 3:**
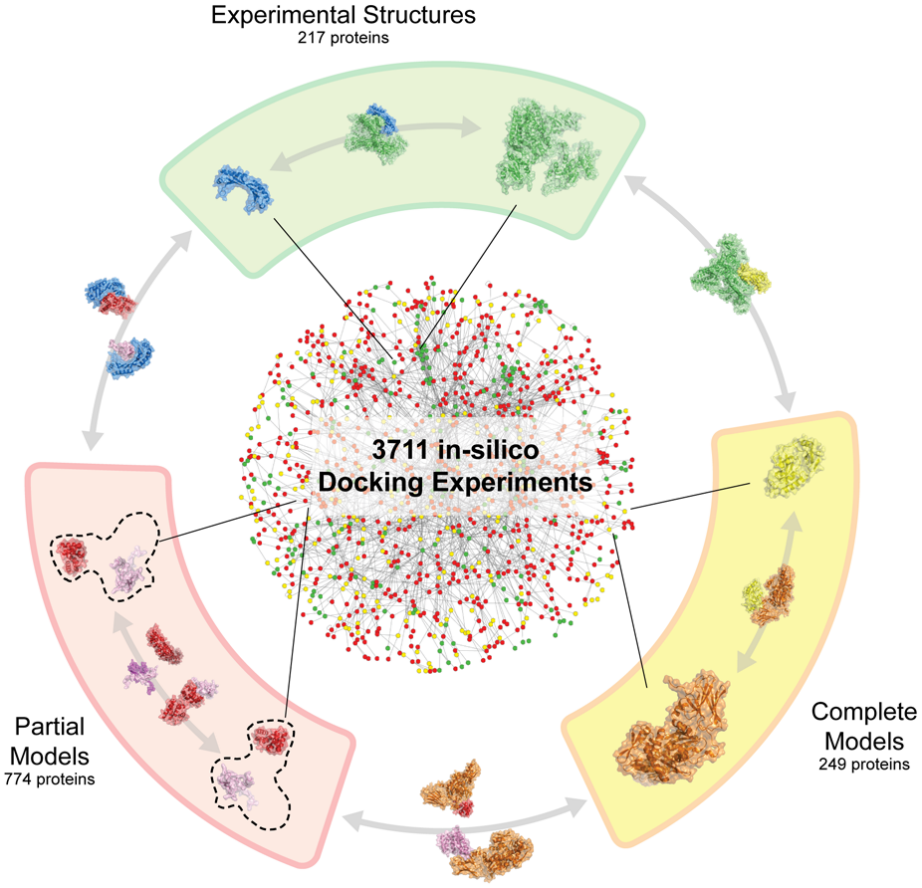
Overview of the high-throughput docking experiment on the yeast interactome. Experimental structures and homology models (complete and partial) were mapped on a high confidence subset of the yeast interactome. Docking experiments were performed for every pair of interacting proteins in order to predict the structure of the binary complex. Green points refer to proteins having experimental structures, yellow points to proteins having complete homology models and red points to the ones having only partial models. Every possible pair of structure types (experimental/experimental, experimental/model, model/model) was present in the experiment.

Altogether, we collected structural information for 1240 proteins involved in 3091 interactions. We then ran docking experiments on each interaction and, for those proteins where we had several partial models we analyzed all the possible combinations. This led to a total number of 3711 docking experiments (Figure 3), of which we successfully completed 3401 (310 failed due to unrecoverable technical reasons), corresponding to 415 homo- and 2986 heterodimers. Of the successful cases, 325 are between two experimentally determined structures, 348 involve a complete homology model and the remaining 2728 involve at least one partial model. As detailed above, all the docking experiments were performed using ZDOCK 3.0, collecting the top 3 solutions, and run on a cluster of IBM Power PC 970MP processors at 2.3 GHz hosted by the Barcelona Supercomputing Center.

To increase the chances of finding an acceptable solution among the top 3 poses and reduce the number of interaction structures to be integrated into the 3D-Repertoire pipeline, we applied the score threshold determined for the benchmark set. Of the 3401 successful experiments 1814 passed the filter, constituting thus a set of higher confidence (see Table S3 in the Supplementary materials for a complete list of the docking experiments).

For 91 out of the 325 docking experiments between experimental structures we could find in the PDB a structure of the interaction itself (see Table S4 in the Supplementary materials). We analyzed separately this set of docking experiments comparing the docking prediction to the real structure of the binary complex. For 37 out of these 91 pairs we could find a high quality solution in the top 3 and for 18 a medium quality one corresponding to a total of 57 good cases, indicating that the success rate on this subset was particularly high. A total of 77 out of the 91 cases had an average score that was above the threshold, and 55 (71%) of those 77 were good cases. These unusually positive results are most likely due to the fact that many experimental structures used for the interacting proteins were extracted from the original complexes (i.e. bound structures), thus simplifying the problem [59].

To further test the performance of the strategy on the yeast interactome, for another 135 of the docking cases we generated an extra prediction of the binary complex by modeling it on a template of interacting homologous proteins, since it has been proved that most homologous pairs do interact in the same way [12]. We then compared the two predictions (docking and homology modeling) and observed an agreement in 42 (31%) cases and, if we analyze only the 95 cases above the threshold, 33 (35%) showed a good agreement. We considered a docking solution to agree with its homology model counterpart if it is classified as acceptable when compared to it. Even if the accordance between a docking prediction and a model build by homology cannot guarantee its correctness, it provides a higher confidence to the predicted conformation.

We also checked whether there is any detectable bias of the docking scores with respect to biophysical nature of the interactions or the experimental techniques that identified them. We classified the interactions into *binary* and *multicomponent* (see the *Methods* section for details) and analyzed the proportion of the two classes in docking cases with scores above the confidence threshold. Table 3 shows how the distribution of the two classes on the larger initial set of interactions and on the higher-confidence set are maintained, which indicates that no particular preference in the success rate of the docking experiments is due to the method used for identifying the interaction.

**Table 3:** Type of the docked interactions.

Interactions	High confidence dataset (13614 interactions)	Successful docking cases (2834 interactions)	Docking cases above the threshold (1661 interactions)
***Binary***	9475	2152	1281
***Multicomponent***	2031	404	236
***In both categories***	785	209	117

Classification of interactions in the large scale docking experiment based on the nature of the experimental method used to identify them.

### Binary interaction between MET30 and RET1

**Figure 4:**
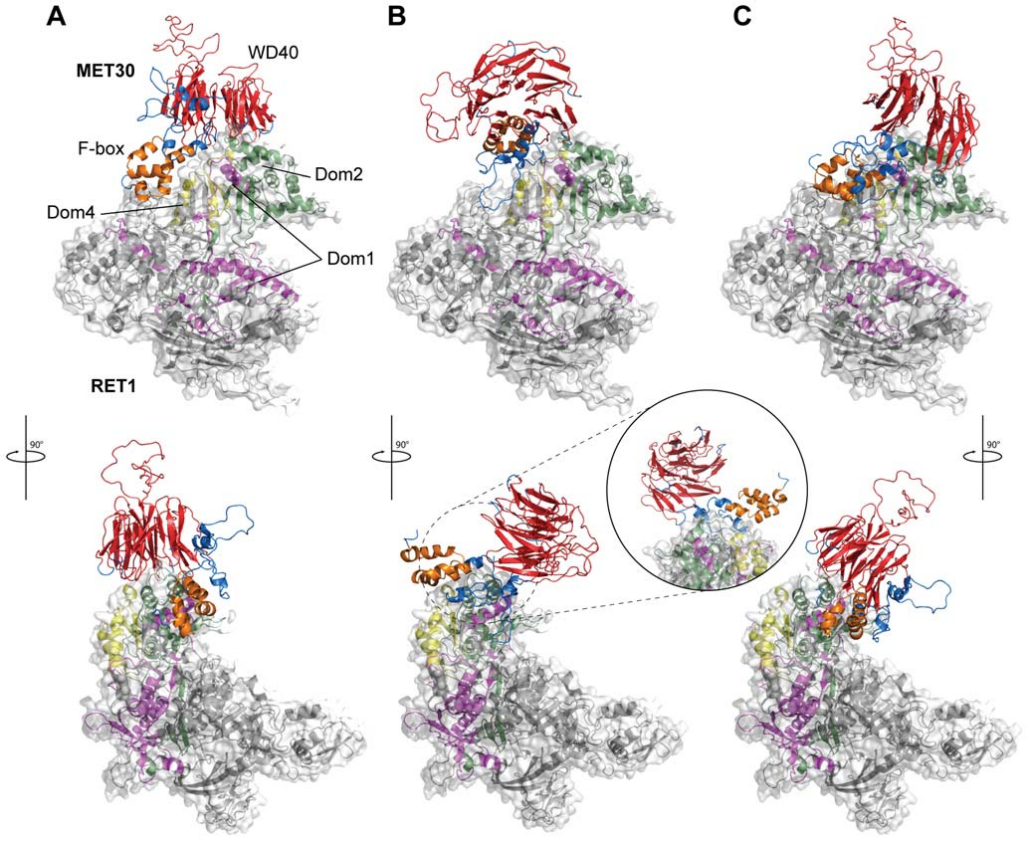
Docking MET30 and RET1, a component of RNA polymerase III. Prediction of the docking between RET1 (grey surface representation) and MET30 (cartoon representation on top). The three top predictions are shown in (A), (B) and (C). Domains of RET 1 are colored in magenta (Dom1), green (dom2) and yellow (dom3) while domains in MET30 are colored in orange (F-box) and red (WD40).

The main objective of this high-throughput docking experiment is to provide molecular details for as many protein-protein interactions as possible in yeast, so that the 3D-Repertoire consortium, and scientific community in general, can benefit from them. For instance, the docking models corresponding to the 1398 binary interactions above the score threshold can be directly used as initial hypothesis to explain the mode of interaction and to design easy mutagenesis experiments to test these hypotheses (see Text S1 and Table S7 in the Supplementary materials for additional information on this topic). An illustrative example is the interaction between MET30 (F-box protein MET30, YIL046w) and RET1 (DNA-directed RNA polymerase III subunit RPC2, YOR207c) that has been reported in two large scale Y2H screens by Uetz el al. [1] and Hazbun et al. [60]. MET30 is a protein localized in the nucleus that is known to control cell cycle function, sulfur metabolism, and methionine biosynthesis as part of the ubiquitin ligase complex, while RET1 is the second largest core component of RNA polymerase III and is proposed to contribute to the polymerase catalytic activity. In the lack of experimental structures for the two proteins, homology models were used. For MET30 the model is based on the structure of a human homolog BTRC (PDB id 1p22) sharing 39% sequence identity with the original protein and including the F-box domain, together with 6 repeats of the WD40 domain (covering 63% of the protein primary sequence). For RET1 the model used is based on the homologous protein in RNA Polymerase II in yeast (PDB id 1i50) and covers the entire sequence of the protein. The docking yielded and average score of the top 3 solutions of 1968.47, which is clearly above the threshold (1386) and was ranked in position 100 in the global list for the complete interactome. In the three predictions generated by the docking program MET30 is shown to interact with RET1 from roughly the same direction but with different orientations (Figure 4). The interaction mainly involves domains 1, 2 and 4 in RET1 while in MET30 the two known domains (F-box and WD40) are involved only in two of the predicted poses. In one of the predictions (Figure 4B) MET30 seems to interact with RET1 only through the linker region. In the lack of additional biological characterization of this particular case, the three poses generated by the docking program suggest a plausible mode of interaction and represent an interesting hypothesis from which it is possible to start further investigations.

### Reconstruction of a trimeric complex

**Figure 5:**
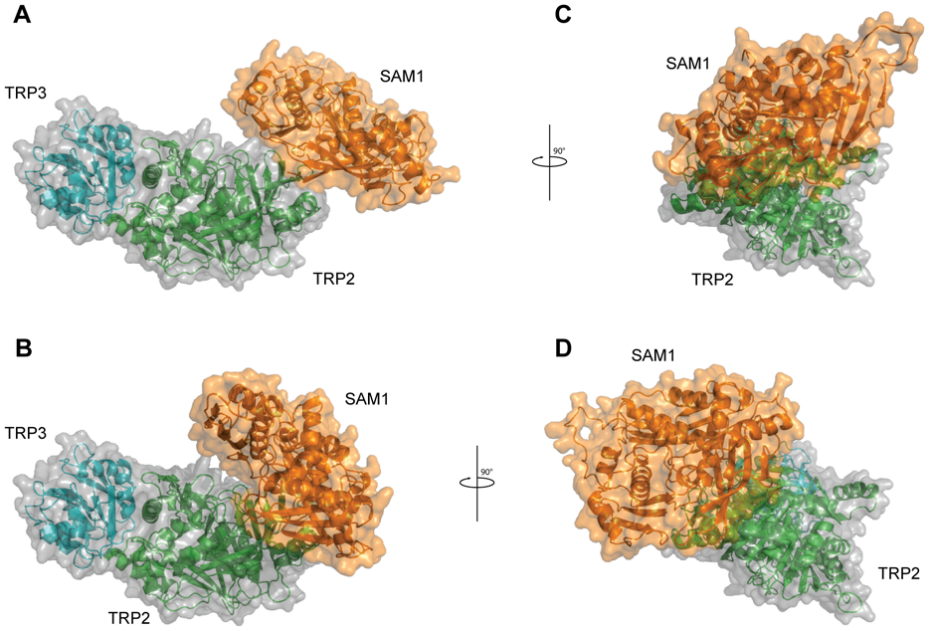
Completing anthranilate synthase complex (TRP2-TRP3) with SAM1. Hypothetical reconstruction of the trimeric complex between TRP2, TRP3 and SAM1. Models for TRP2 and TRP3 were superposed to the template structure of Anthramilate Synthase (PDB id 1qdl) while SAM1 was placed based on the predictions of the docking with TRP2. Two possible conformations are shown in (A,C) and (B,D). The rotation of 90° around axis y in (C) and (D) shows the different orientation of SAM1 with respect to the rest of the complex.

In the frame of the 3D-Repertoire project, the docking predictions generated in this experiment can also be used to complete and complement partial models for several complexes, where some of the interacting subunits can be modeled by homology and the rest are provided by the docking predictions. This is the case, for example, for a complex identified in two large scale experiments using tandem affinity purification techniques (Gavin et al. 2002 [53] and Gavin et al. 2006 [5]). The complex is formed by three components, TRP2 (Anthranilate synthase component 1, YER090w), TRP3 (Anthranilate synthase component 2, YKL211c) and SAM1 (S-adenosylmethionine synthetase 1, YLR180w). None of these yeast proteins have an experimental structure, therefore we collected homology models from ModBase [46]. A structure for the heterodimeric complex between TRPE and TRPG (two homologs of TRP2 and TRP3 respectively) from *Sulfolobus solfataricus* is already present in the PDB (id 1qdl). We could then reconstruct the yeast ternary complex by superposing the model for TRP3 to the structure of TRPG in the crystallographic complex and the model for TRP2 to TRPE. Then we used the results for the docking between TRP2 and SAM1, with an average score of 1717.4 and ranked 398 in the global interactome space, to place the structure of the latter in the complex (Figure 5). All the three predictions for the docking between TRP2 and SAM1 agree on the relative position between the two, placing SAM1 at the opposite end with respect to the interaction interface with TRP3 in the heterodimer (Figure 5). While two of the predictions are almost identical the third shows a slight rotation of SAM1 with respect to TRP3 (Figure 5C and 5D) thus generating two different possibilities for the hypothetical reconstruction of the trimeric complex.

### Concluding remarks

Throughout this manuscript, we have presented a strategy, based on high-throughput docking, to suggest structural details for several thousands of protein-protein interactions in yeast. To do this, it has been necessary to select the best suited state-of-the-art methods in protein docking for implementing a fully automated procedure and to define a score threshold to increase the chances of obtaining a correct model of the interaction. We have also shown that the use of high-quality homology models in docking experiments drastically increases their applicability and coverage of whole interactomes, and it does not seem to imply a critical loss of accuracy with respect to the use of crystal structures, although this cannot be precisely quantified. Finally, we have explored the added value of expert manual intervention and the inclusion of experimental information, when available, in specific docking predictions, showing that these factors do indeed represent a significant improvement in the capacity of docking to produce accurate models.

High-throughout interaction discovery initiatives have permitted to draft the first interactome networks that cover a significant portion of the interaction space in several model organisms. These networks have proved to be very useful for deciphering the underlying regulatory mechanisms of certain cellular processes and pathological pathways [61]. However, their abstract nature implies a limited relationship with physical reality. The real picture of a cell will ultimately come when complete interactomes and pathways can be complemented by a comprehensive repertoire of the 3D structures of protein complexes. This places structural biology, both experimental and computational, in a crucially important position for systems biology. With the second generation structural genomics initiatives being in their production phase, we hope the coming years will see an explosion of structural information for interacting cellular components, which will produce whole-cell framework at atomic-level detail of increasing quality. In this scenario, the scaling up of classical methodologies to predict and model macromolecular complexes to handle thousands of interactions, such as the approach presented here, will become paramount.

## Supporting Information

Text S1 Supplementary information including details on the methods used throughout the work.
(0.12 MB DOC)

Figure S1 Distribution of the sequence identity to the target protein for all the models used in the large scale docking experiment.
(0.15 MB TIF)

Figure S2 Distribution of the average RMSD between models and unbound structures, models and bound structures and bound and unbound structures for the benchmark 3.0. While the average RMSD between models and structures is around 3.4 Å the RMSD between bound and unbound structures is around 1.20 Å.
(0.35 MB TIF)

Figure S3 Ratio between the number of “good” cases (cases having at least one acceptable solution in the top n) and the total number of cases satisfying the threshold for increasing values of the threshold. (A), (C), (E) and (G) refer to ZDOCK 3.0+pyDock while (B), (D), (F) and (H) refer to ZDOCK 3.0 alone. (A) and (B) are relative to the top 1 solution, (C) and (D) to the top 3, (E) and (F) to the top 5 and (G) and (H) to the top 10.
(0.66 MB TIF)

Figure S4 Success rate for the top predictors in the CAPRI experiment. Predictors name can be found in Table S6. The two black stars are indicating respectively the group of Zhiping Weng (ZDOCK) and Juan Fernandez-Recio (pyDock).
(0.39 MB TIF)

Table S1 Number and percentage of cases for which an “at least acceptable” solution is found in the top n (n = 1,3,5,10) for the different docking tools that have been tested.
(0.11 MB DOC)

Table S2 Number of cases for which ZDOCK 3.0 alone and ZDOCK 3.0+pyDock agree. The two programs are considered to agree on a case if there is a pose that is ranked in the top n (n = 1,3,5 or 10) by both the programs. If this pose is at least acceptable then the two programs are considered to agree on a “good” case.
(0.06 MB DOC)

Table S3 Results of the large scale docking experiment. The table reports all the interactions for which it was possible to calculate a docking prediction. The first two columns are the ORF names of the proteins involved in the interaction. The third and fourth columns are the corresponding structures. For experimental structures these fields correspond to the PDB ID and chain, for models the name of the model corresponds to the Swiss-prot id plus an index. Models can be downloaded from http://gatealoy.pcb.ub.es/docking_paper/. The name of the PDB file in the downloadable tarball corresponds to the model name. The fifth and sixth columns indicate if the structures are complete experimental structures, complete models or partial models. The seventh column is the average score of the top 3 solutions, while the eighth column contains YES if the score is above the confidence threshold and NO otherwise. The ninth column is the name of the tarball containing the results (results can be downloaded from http://gatealoy.pcb.ub.es/docking_paper/). The last two columns indicate which one of the two structures is the receptor (chain A in the complex) and which one is the ligand (chain B in the complex).
(0.70 MB XLS)

Table S4 Docking cases in the high-confidence interactome for which there is an experimental structure for the interaction. The format is the same of the one for Table S3. An additional column list the best classification that can be reached by the docking predictions when compared to the experimental structures of the interaction.
(0.03 MB XLS)

Table S5 Distribution of the good cases between the different difficulty levels in the Benchmark 3.0 dataset. The results are referred to the predictions provided by ZDOCK 3.0 alone, without the pyDock rescoring.
(0.07 MB DOC)

Table S6 Success rate for the CAPRI predictors participating in at least 12 targets. Successful targets are those targets for which at least one prediction was classified as acceptable.
(0.12 MB DOC)

Table S7 Results of the simulation of an alanine scanning experiment. Refer to the Supplementary Text S1 for the details.
(0.05 MB XLS)
