# Supplementary material for: Pushing Structural Information into the Yeast Interactome by High-Throughput Protein Docking Experiments
Source: PLoS Comput Biol. 2009 Aug 28;5(8):e1000490. doi: 10.1371/journal.pcbi.1000490 (PMC2722787; doi:10.1371/journal.pcbi.1000490)
Supplement: Text S1 — Supplementary information including details on the methods used throughout the work. (0.12 MB DOC) [file pcbi.1000490.s001.doc]

**Pushing structural information into the yeast interactome by high-throughput protein docking experiments**

Roberto Mosca^1,2^, Carles Pons^2,3^ , Juan Fernández-Recio^1^ and Patrick Aloy^1,2,4^

# 1. Institute for Research in Biomedicine, c/ Baldiri Reixac 10, 08028 Barcelona, Spain.

# 2. Barcelona Supercomputing Center, c/ Jordi Girona 29, 08034 Barcelona, Spain.

# 3. National Institute of Bioinformatics (INB), c/ Jordi Girona 29, 08034 Barcelona, Spain.

# 4. Institució Catalana de Recerca i Estudis Avançats (ICREA), Passeig Lluís Companys 23, 08010 Barcelona, Spain.

# Supplementary materials

**Structural characterization of proteins in the yeast proteome and interactome**

By using the procedure highlighted in the Methods section of the main manuscript it was possible to identify experimental structures for 336 proteins and complete homology models for 441 proteins in the yeast ORFeome. For an additional 1115 proteins only partial models could be found. Of this initial set a subset was also present in the high-confidence (HC) interactome. If we limit to those interactions for which there is structural characterization for both the interactors then for 217 proteins there was an experimental structure, for 249 there was a complete model and for 774 there were partial models.

# Identifying inter chain interactions in a PDB file

In order to identify interacting chains inside a PDB file every pair of chains is analyzed to identify putative residue-residue interactions. Pairs of residues having more than one atom-atom interaction (on different pairs of atoms) are counted only once. Interactions are ordered based on their strength: covalent interactions (disulfide bridges), hydrogen bonds, van der Waals interactions. The strongest interaction type is recorded for every pair of interacting residues. Putative interactions are identified using the following criteria:

- two sulfur atoms of a pair of Cysteines are at a distance less than 2.56 Å (two times the covalent radius of Sulfur plus 0.5 Å) are considered covalently bonded (disulfide bridge);
- all interactions for which there is a pair of atoms that is at a distance less then the sum of the two covalent radii plus 0.5 Å that are not forming a disulfide bridges are considered clashes and are not taken into account in the total number of interactions;
- all pairs N-O, O-N, O-O, N-N for which the distance satisfies the following limits are considered putative hydrogen bonds:

| **Type** | **Max distance** |
| --- | --- |
| O-O | 2.8 Å |
| O-N or N-O | 3.1 Å |
| N-N | 3.2 Å |

- All pairs of atoms that are at a distance that is less than the sum of the two van der Waals radii are considered as a van der Waals interaction.

Two chains are considered as being interacting if there are at least 8 residue-residue interactions of any type between them (clashes are not taken into consideration).

# Homology modeling of binary interactions between proteins with complete structural data

Starting from the sequences of all the 777 ORFs for which we have complete structural information (336 proteins having experimental structure + 441 proteins having a complete homology model) the PDB was searched for structures of homologs to those 777 ORFs. We consider as a homolog every structure for which we have a BLAST alignment having E-value less than or equal to 1e-4, a coverage (number of aligned residues / length of the ORF) higher than or equal to 90% and sequence identity (number of identical residue / length of the alignment including gaps) higher than or equal to 30%.

For every pair of chains, if they belong to the same PDB file and if they have at least 8 interactions (disulfide bridges + hydrogen bonds + van der Waals interactions) they are considered as being interacting (see the previous section).

A total of 749 interactions were identified in 1800 PDBs involving 6621 pairs of chains. Starting from the 749 interactions identified we assign a model or a structure to every ORF involved in an interaction. If the ORF has more than one crystallographic structure, the one with the highest resolution is taken, if the ORF has no crystallographic structures but it has an NMR structure this one is taken, otherwise the homology model for the ORF is used. For every pair of interacting chains in the set of the templates collected with the procedure described before, the structures (or models) for the single ORFs are superposed to the corresponding homologs in the template. Templates showing poor quality (with a number of clashes between the two chains higher than 20) are excluded. A total of 22751 alignments are produced. For every pair of ORFs, multiple models are generated (using different templates). At this point one model is selected for every pair of ORFs for which an experimental structure of the interaction is not already present in the PDB. Part of the models are excluded based on the following conditions:

- generated from templates that are not X-ray or NMR structures
- having an RMSD for the superposition of one of the two components higher than 8 Å
- having a coverage for the structural alignment of one of the two components lower than 75%

The remaining models are filtered and selected using the following criteria:

- The model generated with the template with the highest sequence similarity to the two ORFs is preferred to the others
- If possible homodimeric templates are used to model homodimers and heterodimeric templates are used to model heterodimers
- If more than one model share the same previous conditions then the one with the highest resolution is used
- If more than one model share the same previous conditions then the one maximizing the number of interactions in the modeled complex is used
- If more than one model share the same previous conditions then the one minimizing the number of clashes is used

A total of 559 models are finally selected, 135 of which corresponded to interactions in the high confidence interactome generated for the high-throughput docking experiment.

**Imposing a threshold on the average score of the top n solutions**

In the plots shown in Figure S3 you can see the ratio between the number of “good” cases (cases having at least one acceptable solution in the top n) and the total number of cases satisfying the threshold for different values of the threshold. For pyDock the score is minimized, implying that in order to satisfy the threshold a solution needs to have a score that is less than the threshold. For ZDOCK it is the contrary, the score is maximized and the cases are selected if they are higher than the threshold.

**Analysis of the results of the CAPRI experiment**

The results on the prediction of docking complexes from all the available rounds (Rounds 1-15) of the CAPRI [1-3] experiments were analyzed. Targets 1-28 were included in the analysis with the exclusion of targets 15-17 and 22-23 due to unavailability of the data.

The results for the predictors participating in at least half of the targets (12) were then analyzed in terms of number of targets for which predictions were provided and number of successful targets (having at least one prediction classified as acceptable). The results can be found in Table S6 and in Figure S4. The average success rate of the top 10 performers (ordered based on the success rate itself) is around 46%. We have to remember that every predictor in the CAPRI experiment is allowed to provide up to 10 predictions for every target. Thus the results presented here should be compared to the success rate on the benchmark 3.0 dataset when we take into consideration the top 10 solutions. By using a threshold we can reach a success rate using ZDOCK 3.0 alone of 38% on the benchmark 3.0 (see Figure S3H). We esteem this second percentage in good agreement with the first and even surprisingly high if we consider that the test on benchmark 3.0 is unsupervised and totally automated (while in the CAPRI experiment the cases are analyzed singularly and it is possible to add biological knowledge and refinement steps to the analysis). On the other hand we have to highlight that the benchmark 3.0 contains all the cases of the CAPRI experiments and has probably been used as a test set for the development of the docking methods used in the current work.

As we can see in the chart, ZDOCK (from Weng’s group) and pyDock (from Juan Fernandez-Recio’s group) are amongst the best performing methods in the CAPRI experiments (see the two black stars in Figure S4).

**Simulation of an Alanine Scanning experiment on the benchmark 3.0 dataset**

One possible concern about the results of the docking experiment is related to the extent those result can provide a biologist with useful hypothesis about the interaction between two proteins that can be experimentally verified. One question in this sense could be: “Would the knowledge of the docking predictions for a set of cases improve the probability of an experimentalist to identify residues that are important to the interaction by performing an alanine scanning mutation experiment?”

Given two proteins that are known to interact a possible way to identify where the interacting surfaces lie is to mutate one by one all the residues in the two interacting proteins to an alanine and verify experimentally if the two proteins still interact after the mutation.

Docking predictions could help the experimentalist in focusing on a restricted number of residues to be tested (the residues that are already predicted as being part of the interaction interface) thus saving time and money. But would this approach really help the biologist? And to which extent?

We addressed these questions by simulating the experiment the biologist would do either with the knowledge of the docking solutions or without them to estimate how changes its probability of success when going from one condition to the other.

Several approximations were done in order to be able to perform the simulation and they will be explained in the rest of this section.

We ran the simulation on the hypothesis that the experimentalist knows the structure of the two interacting proteins; in fact, this knowledge is essential in order to be able to apply computational docking methods to produce putative models for the interaction.

In the absence of any information about where the interface lies the experimentalist could try to mutate randomly any residue in the surface of the two proteins until he finds one that appears to disrupt the interaction. We will call this method “*blind*”. If we assume that a mutation of any residue in the interface would result in the suppression of the interaction itself, then the probability of success of a *blind* experiment is given by P_bs_

P_bs_ = N_i_ / N_s_

where N_i_ is the number of residues in the interface and N_s_ is the total number of residue on the surface of the protein. A residue is considered as being on the surface if its relative solvent-accessible surface area (ASA) is higher than 5% and it is considered as being part of the interface if it is on the surface and its loss of ASA after complexation is higher than 1 Å^2^ [4,5].

If the experimentalist is provided with a set of docking predictions he/she could decide to concentrate his efforts on the putative interface residues that are present in the docking solutions. In particular, for example, he could limit the random selection of the residues to mutate to the union of the residues in the interaction interface of the docking predictions. We will call this second approach as “*guided*”. In this case the portion of the sampled residues that identifies a successful hit is given by the intersection with the real set of interface residues.

Our question is: “If we provide the experimentalist with three docking predictions how does change his probability of being successful in identifying any interface residue in its *guided* alanine scanning experiment with respect to the simpler *blind* experiment?”

In order to run the simulation we need to know both the real interaction interface and the docking solutions. Therefore, a good candidate dataset for the simulation is the benchmark 3.0 used throughout our work. In particular we used the subset of the benchmark 3.0 that we have identified as higher confidence subset by the application of a threshold on the average score of the top 3 docking solutions.

For this subset it was possible to calculate the four quantities that were needed in our simulation for both the ligand and the receptor proteins:

- number of residues on the surface (*surface residues*)
- number of residues in the interface (*real interface residues*)
- number of residues in the union of the interfaces presented by the three docking predictions (*sampling residues*)
- number of residues in the intersection between the set of sampling residues and the real interface residues (*intersection residues*)

We calculated all these quantities for both the receptor and the ligand in the 37 cases that compose the higher reliability subset of the Benchmark 3.0 (37 x 2 = 74 cases in total). It was then possible to simulate the outcome of the *blind* and *guided* alanine scanning experiments by doing random samplings without replacement on the set of all the surface residues (blind) or on the residues in the union of the interfaces shown by the docking predictions (guided). An experiment was considered to be successful if at least one interface residue was selected.

We run 1000 experiments for every case using different sampling dimensions (10, 20 and 50 that means doing 10, 20 or 50 alanine mutations). Finally the portion of successful samplings out of the 1000 was taken as an estimation of the probability of success of the experimentalist in the two conditions (see Table S7). We also evaluated the average number of interface residues hit in every experiment.

As we can see, if we do 10 alanine mutations, in more than 85% of the cases the probability of success for the *guided* experiment is higher than the probability of the *blind* experiment. This percentage lowers to 76% when we do 20 alanine mutations. For 50 alanine mutations it is 41% but the value is not statistically significant. We have to take into account the fact that for many cases (47, corresponding to 63% of the total) the number of sampling residues is lower than 50 meaning that, in reality, for the guided experiment we can only do less than 50 alanine mutations making the comparison to the blind experiment in these cases biased in favor to the latter. It is also worth noting that for a few cases there is no intersection between the sampling residues and the real interface residues. The behavior of the success rate for the guided experiment is thus in the case of 50 mutations of type “ON/OFF” (either a success probability of 1.0 or of 0.0) with a great majority of cases where the success rate is 1.0 and a few cases where the success rate is 0.

Since the number of residues in the interface is in general around 10-20% of the entire number of residues in the surface (see the third and fourth columns of Table S7) it is not uncommon to randomly identify at least one of the interface residues when doing 10 or 20 alanine mutations. Doing 50 alanine mutations the probability is particularly high as it is reflected in the success probabilities for the blind experiments in table S7.

For this reason we tried to evaluate if there is a significant difference in the average number of interface residues that can be hit by the two approaches. The average number of interface residues identified in the guided experiment, indeed, is higher than the same number of residues in the blind experiment, for a significant number of cases (around 85% of the cases) for all the sampling dimensions. If we take into consideration the problem of the false negatives this fact represents an advantage for the guided approach compared to the blind approach.

In conclusion, the guided approach shows, in our simulation, to have a higher success rate than the blind approach for a significant number of cases. The average number of interface residues that are sampled is higher for the guided approach than for the blind one indicating a higher probability of discovering an interface residue even in the presence of false negatives.

**Evaluating the significance of the previous statistics**

In order to asses the statistical significance of these results we used bootstrapping for simulating 10000 random sets of experiments (10000 times the experiments on all the 74 cases) in order to estimate the distribution of the number of cases for which a guided experiment could be more successful than a blind one in the null hypothesis that the docking solutions are randomly chosen. To implement this random choice we maintained fixed to the original values the following variables: number of *surface residues*, number of *real interface residues* and number of *sampling residues*. We then evaluate the number of *intersection residues* through a random sampling without replacement in the set of all surface residues of a number of residues equals to the number of *sampling residues*. The number of real interface residues selected during the sampling was used as the “randomized” number of *intersection residues*.

Since we performed 10000 random sets of experiment we can estimate the p-value of the percentages reported in the manuscript with a maxmum resolution of 1.0E-04.

**References**

1. Lensink MF, Mendez R, Wodak SJ (2007) Docking and scoring protein complexes: CAPRI 3rd Edition. Proteins 69: 704-718.

2. Mendez R, Leplae R, De Maria L, Wodak SJ (2003) Assessment of blind predictions of protein-protein interactions: current status of docking methods. Proteins 52: 51-67.

3. Mendez R, Leplae R, Lensink MF, Wodak SJ (2005) Assessment of CAPRI predictions in rounds 3-5 shows progress in docking procedures. Proteins 60: 150-169.

4. Jones S, Marin A, Thornton JM (2000) Protein domain interfaces: characterization and comparison with oligomeric protein interfaces. Protein Eng 13: 77-82.

5. Miller S, Janin J, Lesk AM, Chothia C (1987) Interior and surface of monomeric proteins. J Mol Biol 196: 641-656.
