# Supplementary figures and images for: Pushing Structural Information into the Yeast Interactome by High-Throughput Protein Docking Experiments
Source: PLoS Comput Biol. 2009 Aug 28;5(8):e1000490. doi: 10.1371/journal.pcbi.1000490 (PMC2722787; doi:10.1371/journal.pcbi.1000490)

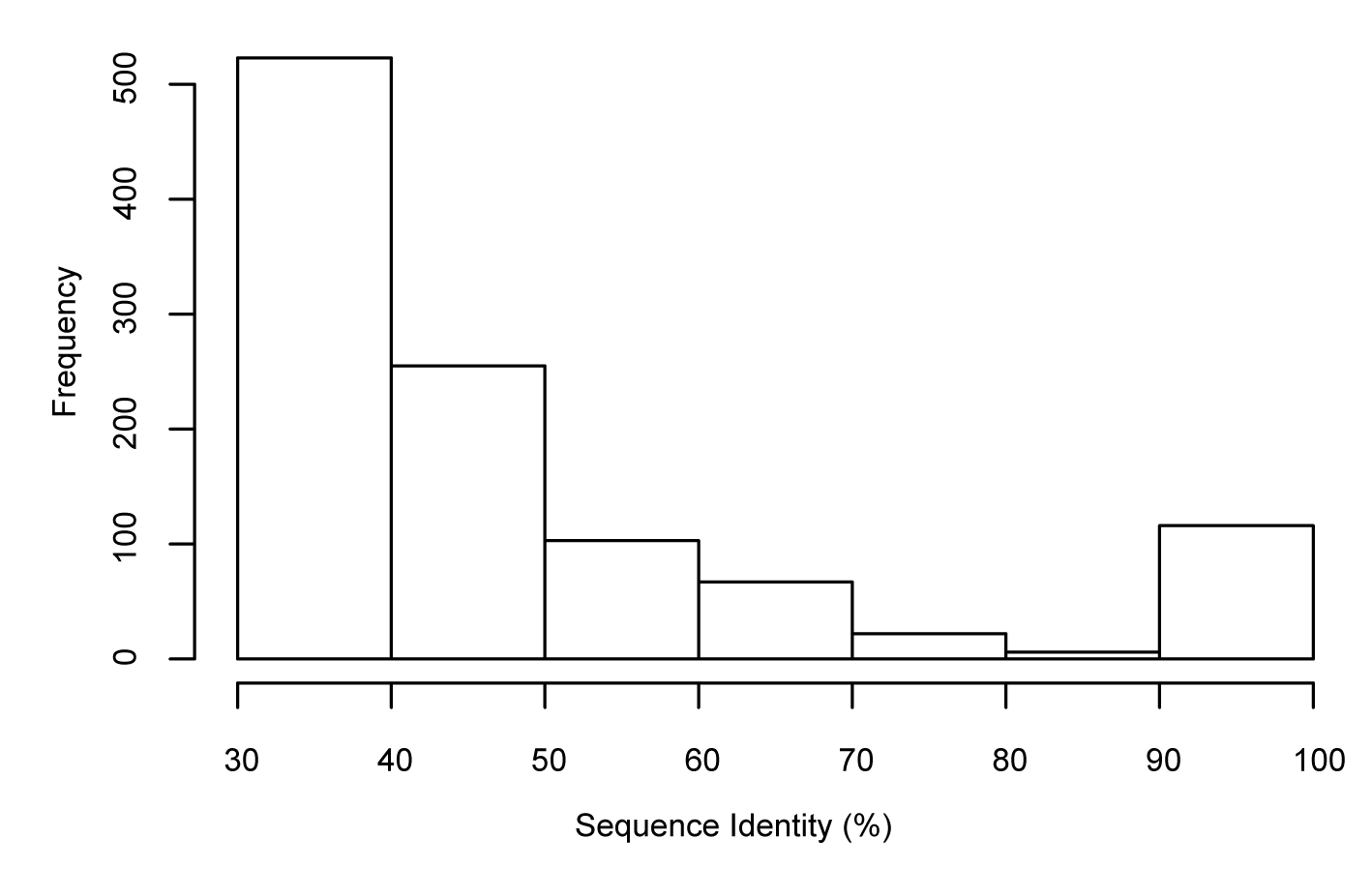

Supplement: Figure S1 — Distribution of the sequence identity to the target protein for all the models used in the large scale docking experiment. (0.15 MB TIF) [file pcbi.1000490.s002.tif]

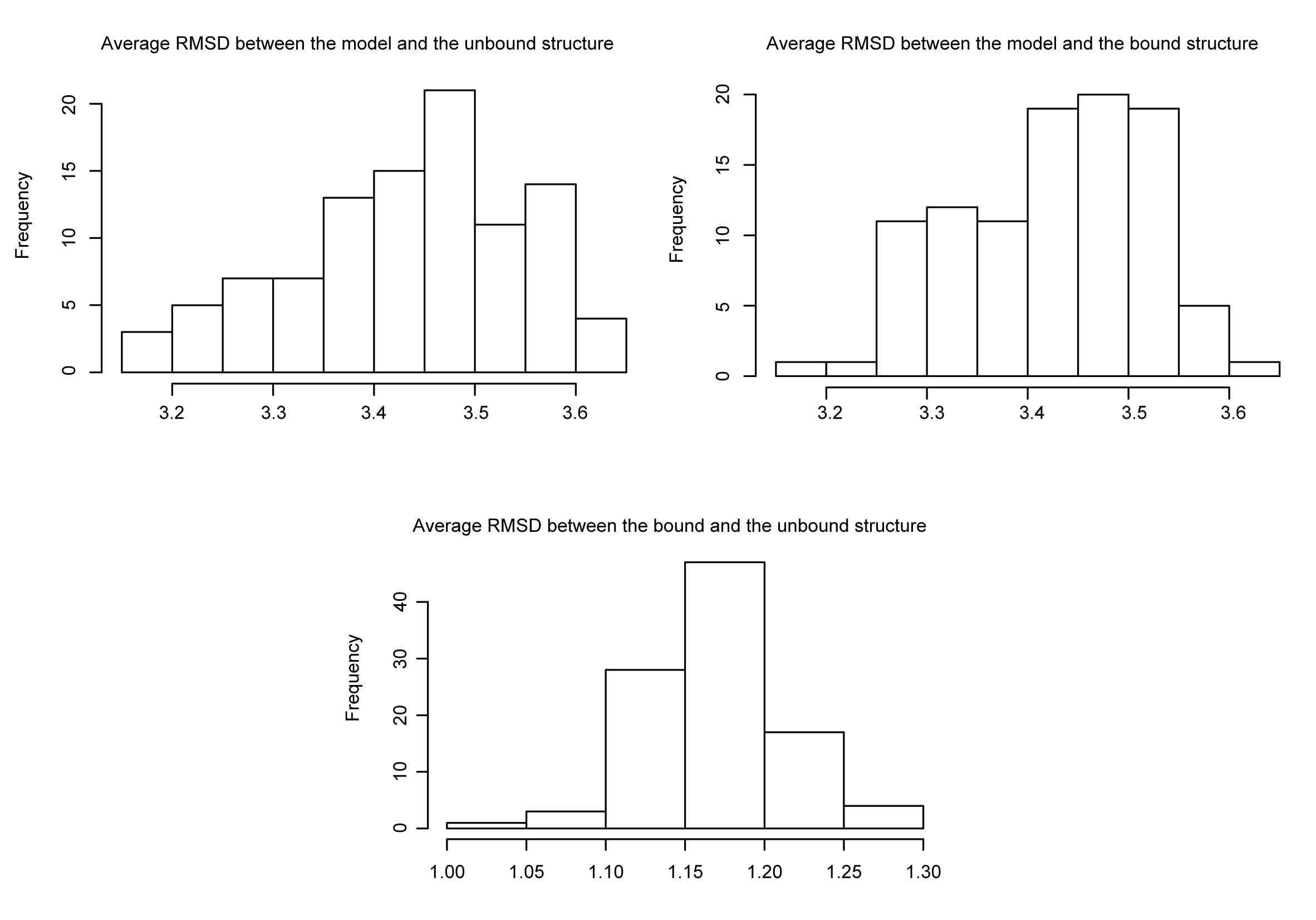

Supplement: Figure S2 — Distribution of the average RMSD between models and unbound structures, models and bound structures and bound and unbound structures for the benchmark 3.0. While the average RMSD between models and structures is around 3.4 Å the RMSD between bound and unbound structures is around 1.20 Å. (0.35 MB TIF) [file pcbi.1000490.s003.tif]

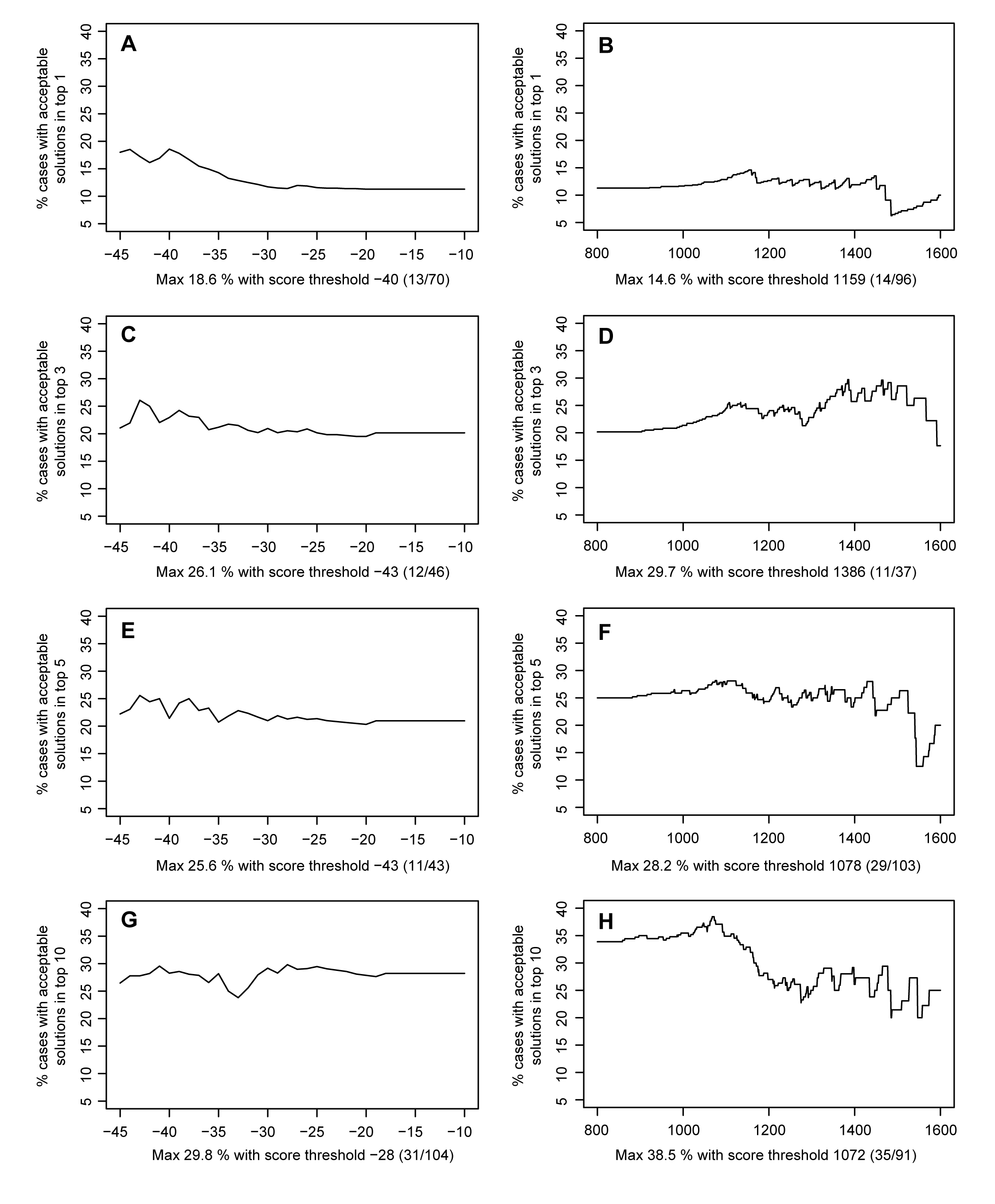

Supplement: Figure S3 — Ratio between the number of “good” cases (cases having at least one acceptable solution in the top n) and the total number of cases satisfying the threshold for increasing values of the threshold. (A), (C), (E) and (G) refer to ZDOCK 3.0+pyDock while (B), (D), (F) and (H) refer to ZDOCK 3.0 alone. (A) and (B) are relative to the top 1 solution, (C) and (D) to the top 3, (E) and (F) to the top 5 and (G) and (H) to the top 10. (0.66 MB TIF) [file pcbi.1000490.s004.tif]

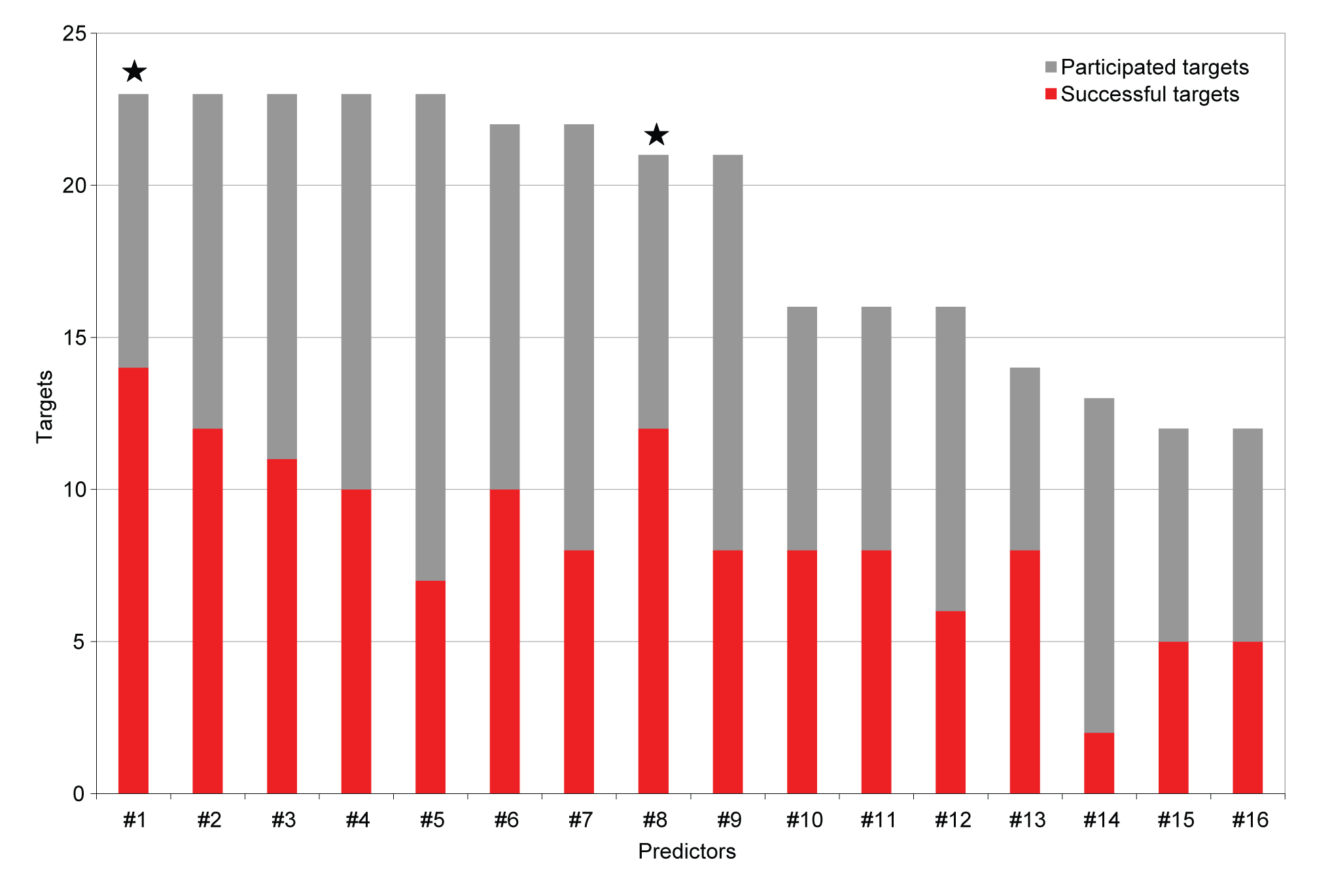

Supplement: Figure S4 — Success rate for the top predictors in the CAPRI experiment. Predictors name can be found in Table S6. The two black stars are indicating respectively the group of Zhiping Weng (ZDOCK) and Juan Fernandez-Recio (pyDock). (0.39 MB TIF) [file pcbi.1000490.s005.tif]
