# Supplementary material for: Pushing Structural Information into the Yeast Interactome by High-Throughput Protein Docking Experiments
Source: PLoS Comput Biol. 2009 Aug 28;5(8):e1000490. doi: 10.1371/journal.pcbi.1000490 (PMC2722787; doi:10.1371/journal.pcbi.1000490)
Supplement: Table S1 — Number and percentage of cases for which an “at least acceptable” solution is found in the top n (n = 1,3,5,10) for the different docking tools that have been tested. (0.11 MB DOC) [file pcbi.1000490.s006.doc]

**Table S1**

| **Program** | **Top1** | | **Top3** | | | | **Top5** | | **Top10** | | **Tot al # cases** |
| --- | --- | --- | --- | --- | --- | --- | --- | --- | --- | --- | --- |
| FTDOCK | 0 | 0.0% | | 1 | 0.8% | 1 | | 0.8% | 1 | 0.8% | 124 |
| FTDOCK+pyDock | 5 | 4.0% | | 11 | 8.9% | 13 | | 10.5% | 18 | 14.5% | 124 |
| ZDOCK2.3 | 5 | 4.0% | | 9 | 7.3% | 12 | | 9.7% | 18 | 14.5% | 124 |
| ZDOCK2.3+pyDock (vdW) | 9 | 7.3% | | 21 | 16.9% | 31 | | 25.0% | 36 | 29.0% | 124 |
| ZDOCK3.0 | 14 | 11.3% | | 25 | 20.2% | 31 | | 25.0% | 42 | 33.9% | 124 |
| ZDOCK3.0+PyDock (No VdW) | 14 | 11.3% | | 25 | 20.2% | 26 | | 21.0% | 35 | 28.2% | 124 |
| *DOCK+pyDock | 14 | 11.3% | | 25 | 20.2% | 26 | | 21.0% | 35 | 28.2% | 124 |
