# Supplementary material for: Pushing Structural Information into the Yeast Interactome by High-Throughput Protein Docking Experiments
Source: PLoS Comput Biol. 2009 Aug 28;5(8):e1000490. doi: 10.1371/journal.pcbi.1000490 (PMC2722787; doi:10.1371/journal.pcbi.1000490)
Supplement: Table S2 — Number of cases for which ZDOCK 3.0 alone and ZDOCK 3.0+pyDock agree. The two programs are considered to agree on a case if there is a pose that is ranked in the top n (n = 1,3,5 or 10) by both the programs. If this pose is at least acceptable then the two programs are considered to agree on a “good” case. (0.06 MB DOC) [file pcbi.1000490.s007.doc]

**Table S2**

|  | **# cases for which the two programs agree** | **# “good” cases on which the two programs agree** |
| --- | --- | --- |
| **Top 1** | 3 | 0 |
| **Top 3** | 9 | 1 |
| **Top 5** | 21 | 3 |
| **Top 10** | 48 | 6 |
