# Supplementary material for: Pushing Structural Information into the Yeast Interactome by High-Throughput Protein Docking Experiments
Source: PLoS Comput Biol. 2009 Aug 28;5(8):e1000490. doi: 10.1371/journal.pcbi.1000490 (PMC2722787; doi:10.1371/journal.pcbi.1000490)
Supplement: Table S5 — Distribution of the good cases between the different difficulty levels in the Benchmark 3.0 dataset. The results are referred to the predictions provided by ZDOCK 3.0 alone, without the pyDock rescoring. (0.07 MB DOC) [file pcbi.1000490.s010.doc]

**Table S5**

| **Have an at least acceptable solution in…** | **Rigid-body** | **Medium** | **Difficult** | **Total** |
| --- | --- | --- | --- | --- |
| **Top 1** | 14 | 0 | 0 | 14 |
| **Top 3** | 23 | 2 | 0 | 25 |
| **Top 5** | 29 | 2 | 0 | 31 |
| **Top 10** | 36 | 4 | 2 | 42 |
