# Supplementary material for: Pushing Structural Information into the Yeast Interactome by High-Throughput Protein Docking Experiments
Source: PLoS Comput Biol. 2009 Aug 28;5(8):e1000490. doi: 10.1371/journal.pcbi.1000490 (PMC2722787; doi:10.1371/journal.pcbi.1000490)
Supplement: Table S6 — Success rate for the CAPRI predictors participating in at least 12 targets. Successful targets are those targets for which at least one prediction was classified as acceptable. (0.12 MB DOC) [file pcbi.1000490.s011.doc]

**Table S6**

| **#** | **Name** | **Target participated** | **Successful targets** | **% success rate** |
| --- | --- | --- | --- | --- |
| #1 | Weng | 23 | 14 | 61% |
| #2 | Camacho | 23 | 12 | 52% |
| #3 | Wolfson/Nussinov | 23 | 11 | 48% |
| #4 | Eisenstein | 23 | 10 | 43% |
| #5 | Ritchie | 23 | 7 | 30% |
| #6 | Baker | 22 | 10 | 45% |
| #7 | Ten Eyck | 22 | 8 | 36% |
| #8 | Fernandez-Recio/Abagyan | 21 | 12 | 57% |
| #9 | Gray | 21 | 8 | 38% |
| #10 | Sternberg | 16 | 8 | 50% |
| #11 | Bates | 16 | 8 | 50% |
| #12 | ClusPro | 16 | 6 | 38% |
| #13 | Bonvin | 14 | 8 | 57% |
| #14 | Palma/Krippahl | 13 | 2 | 15% |
| #15 | Vakser | 12 | 5 | 42% |
| #16 | Zacharias | 12 | 5 | 42% |
